# Recent Advances in Paper Conservation Using Nanocellulose and Its Composites

**DOI:** 10.3390/molecules30020417

**Published:** 2025-01-19

**Authors:** Mei Jiang, Jingjing Yao, Qiang Guo, Yueer Yan, Yi Tang, Yuliang Yang

**Affiliations:** 1 Institute for Preservation and Conservation of Chinese Ancient Books, Fudan University Library, Fudan University, 220 Handan Road, Shanghai 200433, China; 23110820002@m.fudan.edu.cn (M.J.); 24110820002@m.fudan.edu.cn (Q.G.); yulyang1952@163.com (Y.Y.); 2 Shanghai Institute of Quality Inspection and Technical Research, 900 Jiang Yue Road, Shanghai 201114, China; yaojj@fudan.edu.cn; 3 Shanghai Key Laboratory of Molecular Catalysis and Innovative Materials, Department of Chemistry, Fudan University, 2005 Songhu Road, Shanghai 200438, China

**Keywords:** nanocellulose, composite materials, conservation, paper-based cultural relics

## Abstract

Paper-based cultural relics experience aging and deterioration during their long-term preservation, which poses a serious threat to their lifetime. The development of conservation materials with high compatibility and low intervention has been expected to extend the lifetime of paper artifacts. As a new type of biological macromolecule, nanocellulose has been extensively utilized in paper conservation, attributed to its excellent paper compatibility, high optical transparency, outstanding mechanical strength, and large specific surface area with abundant hydroxyl groups. This review systematically summarizes the latest development of three kinds of nanocellulose (cellulose nanofibril, cellulose nanocrystal, and bacterial nanocellulose) and their composites used for the multifunctional conservation of paper relics. Owing to the strong hydrogen bond interactions between hydroxyls of nanocellulose and paper fibers, nanocellulose can effectively consolidate paper without adding adhesives. The composite of nanocellulose with other functional materials greatly expands its application scope, and the superior performance has been emphasized in paper deacidification, consolidation, antimicrobial effect, antioxidation, UV resistance, self-cleaning, promotion of printing property, reduction in air permeability, and flame retardancy. The application characteristics and future prospects of nanocellulose composites are highlighted in the conservation of paper-based cultural relics.

## 1. Introduction

Paper-based cultural relics, in the forms of ancient books, documents, archives, and paintings, have recorded the development of history, culture, science, and technology of mankind [1,2]. However, due to some endogenous and exogenous factors, paper relics are damaged to varying degrees, which poses a serious threat to the inheritance of human cultural heritages [3].

The main component of paper is cellulose, along with a certain amount of hemicellulose and lignin [4,5]. Paper deterioration is caused by a series of chemical reactions such as acidic hydrolysis, alkali degradation, and oxidative degradation, leading to a decrease in cellulose degree of polymerization (DP) and an increase in chromophore concentration, and macroscopically paper becomes brittle and yellowing [6,7]. It is an urgent and challenging task for libraries, archives, and museums to protect paper relics and pass them to the future generations. In the past, the traditional restoration always depended on the experience of conservators and lacked scientific support. Nowadays, the use of modern technology becomes a new direction in cultural heritage conservation.

Among them, nanotechnology has been widely used in the field of paper conservation and achieved good results. For example, Fistos et al. [8] reviewed the developments of inorganic nanomaterials like magnesium oxide (MgO), calcium hydroxide (Ca(OH)_2_), and zinc oxide (ZnO) utilized for conserving heritage materials such as wood, paper, and textiles. The application of inorganic nanomaterials can enhance the mechanical and esthetic characteristics of artifacts, protect the objects from acid or UV degradation, and provide antimicrobial properties to avoid fungi- or bacteria-induced biodegradation. Zhu et al. [9] summarized the synthesis and application of nano Ca(OH)_2_ in the paper deacidification processes. Baglioni et al. [10,11] showed that the colloidal earth-alkaline hydroxides and carbonates in organic solvents are to be employed for paper pH adjusting. They also overviewed the contributions of colloid and materials science to the art conservation field, including magnesium and calcium hydroxides nanoparticles for the deacidification of paper, canvas, and wood [12]. Franco-Castillo et al. [13] concluded that nanoparticles like silver (Ag), titanium dioxide (TiO_2_), ZnO, MgO, graphene, and layered double hydroxides (LDHs) can be used to handle microbial colonization on heritage substrates. Besides, Gîfu et al. [14] presented the use of layer-over-layer deposited polyelectrolytes in the cleaning, consolidation, and preservation of cultural artifacts, including those composed of organic materials.

**Scheme 1:**
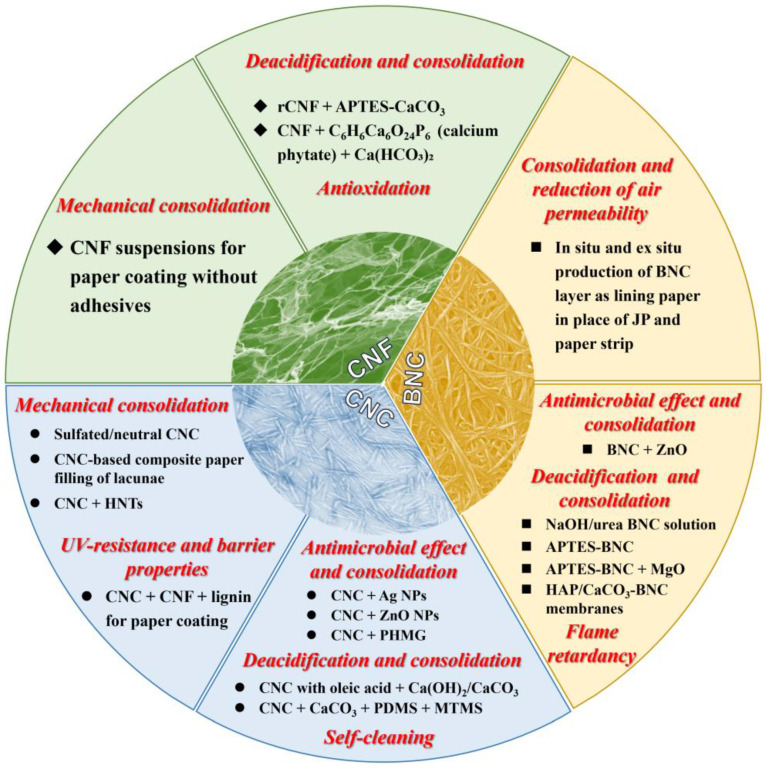
Application of nanocellulose in the conservation of paper-based cultural relics. (Abbreviations: CNF: cellulose nanofibril; CNC: cellulose nanocrystal; BNC: bacterial nanocellulose; APTES: aminopropyltriethoxysilane; PDMS: polydimethylsiloxane; MTMS: methyltrimethoxysilane; HAP: hydroxyapatite; HNTs: halloysite nanotubes; PHMG: polyhexamethylene guanidine; rCNF: reduced CNF; JP: Japanese paper; NPs: nanoparticles).

Considering the requirement of safety, reversibility, and minimum intervention in cultural relics conservation, the compatibility between protective materials and paper substrate should be emphasized. Nanocellulose with good paper compatibility, high optical transparency, and superior mechanical strength has become a promising nanomaterial in recent years. The preparation and application of nanocellulose with improved properties are developing continuously. Moreover, nanocellulose-based materials arouse growing interest from researchers in the field of cultural heritage conservation. Fornari et al. [15] offered a comprehensive overview of how nanocellulose can be utilized to maintain the integrity of cultural heritage such as paintings, wood, and historical papers. Marques et al. [16] reviewed the use of nanocellulose in the restoration of paper documents in chronological order and regarded the use of nanocellulose as an alternative to conventional restoration methods. However, a systematic review on the application of nanocellulose and its composites for the multifunctional protection of paper-based relics is still lacking. Herein, our review briefly introduces the preparation methods and structural properties of three types of nanocellulose, that is, cellulose nanofibrils (CNFs), cellulose nanocrystals (CNCs), and bacterial nanocellulose (BNC). Then, we systematically discuss the use of CNFs, CNCs, and BNC, as well as their composites for paper conservation, such as deacidification, reinforcement and consolidation, antimicrobial effect, antioxidation, UV resistance, self-cleaning, promotion of printing property, reduction in air permeability, and flame retardancy (as shown in Scheme 1). Finally, this review prospects the future requirement and development of nanocellulose composites for paper conservation.

## 2. Classification, Preparation, and Properties of Nanocellulose

**Figure 1:**
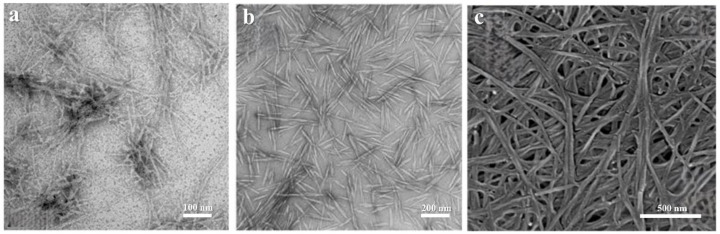
Transmission electron microscope (TEM) image of CNFs (**a**) [19] (reproduced with permission from Industrial Crops & Products; published by Elsevier, 2018). TEM image of CNCs (**b**) [20] (reproduced with permission from Cellulose; published by Springer Nature, 2015). Scanning electron microscope (SEM) image of BNC (**c**) [21] (reproduced with permission from ACS Nano; published by American Chemical Science, 2012).

**Figure 2:**
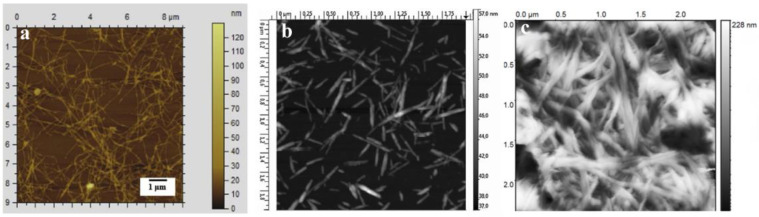
Atomic force microscopy (AFM) image of CNFs (**a**) [22] (reproduced with permission from Carbohydrate Polymers; published by Elsevier, 2020), CNCs (**b**) [23] (reproduced with permission from Journal of Polymers and the Environment; published by Springer Nature, 2017), and BNC (**c**) [24] (reproduced with permission from Molecules; published by MDPI, 2022).

Cellulose, as the most abundant and renewable polymer in nature, has many advantages, such as low cost, non-toxicity, and high biodegradability. Recently, nanocellulose with nanometer size in a certain dimension has attracted widespread attention due to its excellent properties. It has unique nanoscale effects, as well as high purity, high DP, outstanding crystallinity, a large aspect ratio, excellent strength, a fine Young’s modulus, high hydrophilicity, and abundant surface hydroxyls for chemical modification [17]. Moreover, nanocellulose derived from sustainable resources is compatible with the substrate of paper artifacts. Based on its morphology, source, and preparation method, nanocellulose has been classified into three major groups: cellulose nanofibrils (CNFs), cellulose nanocrystals (CNCs), and bacterial nanocellulose (BNC) [18]. The morphology features of three kinds of nanocellulose are shown in Figure 1 and Figure 2 [19,20,21,22,23,24].

### 2.1. CNFs

**Figure 3:**
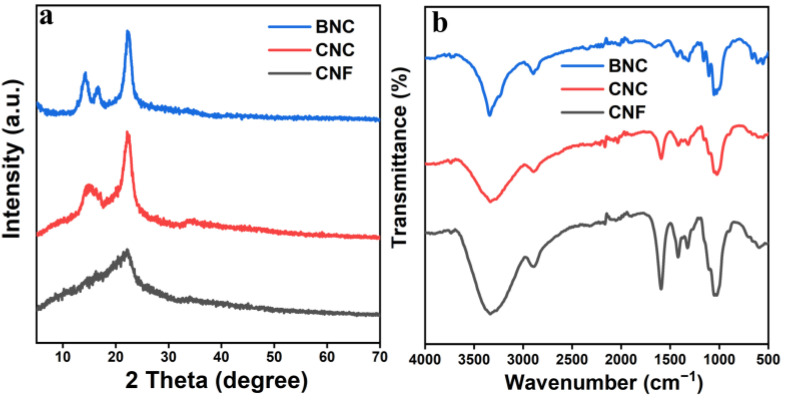
X-ray diffraction (XRD) patterns (**a**) and Fourier-transform infrared (FTIR) spectra (**b**) of CNFs, CNCs, and BNC.

CNFs, also known as nanofibrillated cellulose, microfibrillated cellulose, or nanofibrous cellulose, usually refer to long, flexible, and fibril-like nanofibrils (Figure 1a and Figure 2a) [19,22]. With a wide size range and large aspect ratio, CNF has a nanoscale diameter (10–100 nm) and a micrometer length. Its specific surface area is at least 10 times larger than that of common plant fiber. CNFs consist of both crystalline and amorphous regions, and the crystallinity is usually less than 50% [15]. XRD patterns show that the diffraction peak of CNFs at 2θ of 22.5° is significantly weaker than that of CNCs and BNC, which confirms the low crystallinity of CNFs (Figure 3a). FTIR spectra of CNFs, CNCs, and BNC show the characteristic absorption of cellulose macromolecules (Figure 3b). It can be seen that CNFs have stronger bands at 3330 cm^−1^ for the O-H stretching vibration of H-bonded hydroxyl groups and around 1620 cm^−1^ for the bending vibration of adsorbed H_2_O molecules. CNFs form a porous structure in an aqueous environment, enabling the formation of hydrogels due to the flexible fiber network that facilitates extensive inter-fiber interactions [25]. High-pressure homogenization, cryo-crushing, and grinding are some of the commonly used methods to extract CNFs [18]. These methods can attain cellulose from a variety of lignocellulosic bioresources (e.g., plants and crops) through top-down approaches. However, due to the existence of strong hydrogen bonding between cellulose molecules, the mechanical treatment is energy- and time-consuming. Therefore, combining mechanical techniques with enzymatic or chemical pretreatments can serve as a more energy-efficient alternative [26]. The most common pretreatment methods are enzymatic hydrolysis, 2,2,6,6-tetramethylpiperidine-1-oxyl (TEMPO)-mediated oxidation, carboxymethylation, cationization, and ionic liquid/deep eutectic solvent treatments.

### 2.2. CNCs

CNCs, which are also often referred to as cellulose nanowhiskers, nanorods, nanoparticles, and microcrystals, are rigid rod-like particles with a diameter of 5–70 nm and a length of 100–250 nm (Figure 1b and Figure 2b) [20,23]. CNCs have a high aspect ratio, low density, and high crystallinity, as well as a reactive surface with hydroxy groups that can be grafted by chemical species to achieve different surface properties [27]. The crystal structure of CNCs is cellulose type I, and the degree of crystallinity in CNCs ranges from 54 to 88% [28]. The XRD pattern of CNCs shows the diffraction peaks of 2θ at 14.9°, 16.7°, and 22.5°, representing the crystallographic planes of (1$\bar{1}$0), (110), and (200), respectively (Figure 3a). CNC suspensions can spontaneously assemble into chiral nematic liquid crystals at the nanoscale upon reaching a critical concentration [29]. Due to their excellent dispersion properties, CNCs can undergo targeted self-assembly and more complex modification compared to CNFs [25]. CNCs are also typically gained via top-down approaches from lignocellulosic biomass. The most common preparation process of CNCs is acid hydrolysis. Various strong acids, such as sulfuric (H_2_SO_4_), hydrochloric (HCl), and phosphoric (H_3_PO_4_), can preferentially break down the amorphous regions of cellulose by cleaving the glycosidic bonds between the glucose units, leading to the formation of nanocrystals [30]. Generally, CNCs prepared by HCl hydrolysis are uncharged and electrically neutral. While H_2_SO_4_ or H_3_PO_4_ hydrolysis introduces sulfate or phosphate groups to CNCs, providing better dispersion properties due to electrostatic repulsion. Enzymatic hydrolysis is also used to obtain CNCs by using cellulases to hydrolyze the amorphous region and preserve the crystalline region. Compared with acid hydrolysis, enzymatic hydrolysis is much milder, and it can reduce the defects of the obtained CNCs. The application of enzymatic hydrolysis is sometimes limited due to its high cost, long incubation time, low production efficiency, and poor dispersion of as-prepared CNCs.

### 2.3. BNC

BNC is also considered bacterial cellulose, microbial cellulose, or bio-cellulose. BNC has a diameter of 20–100 nm and a length in micrometer scale, forming a reticulated structure of a nanofiber-weaved three-dimensional (3D) network (Figure 1c and Figure 2c) [21,24]. The crystal structure of BNCs is cellulose type I. Compared with CNFs and CNCs, BNCs have higher crystallinity of 80–90% (Figure 3a) and purity (free of lignin, hemicellulose, pectin, and other biogenic components). The unique structure of BNC endows it with distinct properties such as large surface area, high water-holding capacity, excellent permeability, high wet tensile strength, flexibility, elasticity, and durability [31]. BNC has a peculiar capability to self-assemble fibrils into nanostructured materials [32]. Generally, BNC is synthesized by a bottom-up route. The nanofibers are secreted and produced by some aerobic bacteria using low-molecular-weight sugars (e.g., glucose, fructose, arabinose, sucrose, etc.). *Gluconacetobacter xylinus* is the most efficient producer of BNC, while *Agrobacterium*, *Pseudomonas*, *Rhizobium*, and *Sarcina* are some other bacterial species that are able to produce cellulose as well [33]. Under artificial control, the bacterial strains can produce BNC with different sizes and structures, which makes it easy to achieve industrial production and commercialization.

## 3. Nanocellulose for Paper Conservation

### 3.1. Paper Degradation and Conservation

The natural aging and deterioration of paper relics are spontaneous and irreversible. The degradation of paper involves various chemical reactions like acid hydrolysis, alkaline degradation, and oxidative degradation. The most common degradation process is acid-catalyzed hydrolysis by cleaving the β-1,4-glycosidic bond of cellulose, which results in the decrease in cellulose DP and a significant loss of paper mechanical strength [6]. Moreover, the cleavage of glycosidic bonds also leads to the formation of reduced end groups, which makes cellulose more susceptible to oxidation reactions. Acids in paper come from a wide range of sources, such as the acidic small molecules produced by paper degradation reactions, the fillers (e.g., alum-rosin), pigments and inks, air pollutants, volatile organic compounds (VOCs), and solid particulate matter during storage [34].

The alkaline degradation of cellulose in paper can be divided into alkaline hydrolysis and peeling reaction. The alkaline hydrolysis of cellulose requires high temperature and strong alkalinity, and the reaction conditions are harsh. Alkaline hydrolysis can break some of the glucoside bonds of cellulose and reduce the DP of cellulose. The peeling reaction of cellulose can be carried out under mild alkaline conditions. The nature of the peeling reaction is a β-elimination reaction, in which the reductive end groups of cellulose are shed one by one.

Along with acid hydrolysis and alkaline degradation, paper undergoes oxidative degradation as well [7]. With the aid of light or transition metal species, the oxygen can be converted into reactive oxygen species (ROSs) to trigger the oxidation reaction of cellulose [35,36]. The reductive groups, such as hydroxyls and terminal carbonyls, of cellulose can be oxidized to derivative aldehyde, ketone, and carboxyl groups, and some of them have unsaturated conjugated structures. Moreover, oxidative reaction also leads to the cleavage of cellulose glycosidic bonds through oxygen radicals grabbing hydrogen atoms from the carbon of glycosidic bonds and then the electron migration processes. Therefore, oxidative degradation leads to a decrease in cellulose DP and an increase in chromophore concentration, and macroscopically causes paper yellowing and brittleness.

Additionally, paper relics are also subject to biological attacks by microorganisms, including fungi and bacteria [37,38]. These fungi and bacteria utilize the various organic matters in paper as the source of nutrients to survive and reproduce. The metabolism of mold will secrete a variety of organic acids on paper surfaces, which accelerates the acidic hydrolysis of cellulose [39]. Besides, microbial activity will appear yellow, yellow-brown, and rust-colored foxing [40]. These spots not only affect the beauty and readability of paper artifacts but also make the paper tend to be more brittle. The foxing will continue to spread over time and even infiltrate into the following pages, resulting in an incalculable loss of the value of paper collections.

The target of conservation is to slow down the degradation rate of substrates and prolong the lifetime of artworks. According to the mechanisms of paper degradation, the main paper conservation methods include consolidation, deacidification, antioxidation, cleaning, and sterilization. Recently, nanocellulose has been an innovative material in paper conservation. Up to now, nanocellulose and its composites have been used for paper deacidification, reinforcement and consolidation, antimicrobial effect, antioxidation, UV resistance, self-cleaning, promotion of printing property, reduction in air permeability, and flame retardancy. Table 1 summarizes the application of CNFs, CNCs, and BNC and their composites for the multifunctional conservation of paper, as well as their current and long-term conservation effects [41,42,43,44,45,46,47,48,49,50,51,52,53,54,55,56,57,58,59,60,61,62].

### 3.2. Application of CNFs in the Conservation of Paper-Based Cultural Relics

CNFs can be used as reinforcing agents or additives in the papermaking process to improve the mechanical strength and printing performance of paper [63,64]. The addition of CNFs into paper pulp presents several advantages. For example, CNFs will maintain paper’s original properties and extend paper’s lifetime without damaging the overall structure of paper fibers. CNFs can work as dry strength additives in poorly bonded sheets made of mechanical, recycled, and unbeaten chemical pulps [65]. Typically in aqueous suspension form, CNFs can be introduced into the pulp mixture at the papermaking process or applied as coatings on the paper surface. Ahola et al. [66] found that the tensile strength of paper increased to twice the value by adding 6% of CNFs into the fines-free, slightly beaten softwood pulp. According to Eriksen et al. [67], the degree of fibrillation of CNFs at a specific level increased the paper’s tensile strength. CNFs with high specific surface area enhanced the bonding ability between fibers and consolidated paper structure.

**Figure 4:**
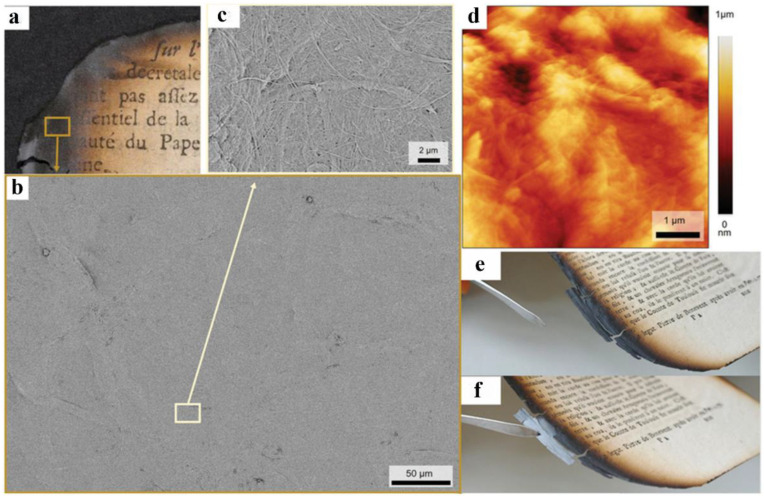
CNF-treated paper. A slight white CNF veil is visible in the medium- and high-damage areas (**a**). Scanning electron micrographs show a completely CNF-coated surface (**b**). CNFs settle into the pores and between the paper fibers and surround them (**c**). AFM surface topography image of a damaged region after the application of CNF (**d**). The CNF layer stabilizes the damaged paper (**e**,**f**) [42] (reproduced with permission from Small; published by Wiley, 2022).

As a paper coating material, CNFs can form strong, transparent, and fibrillar networks with the paper matrix to stabilize paper [68,69]. Völkel et al. [41] applied CNF suspensions to stabilize and reinforce historical paper without adding adhesives. It is an innovative paper stabilization method that eliminates the side effects caused by adhesives. During the coating with CNFs, only one step was feasible, that is, to brush from the left to the right at an angle of 90° onto the paper surface, avoiding the quick formation of fiber agglomerations. After coating of CNFs, no clumping was formed, and the damaged area of the paper was consolidated. The treatment of historical paper with CNF suspensions not only provides significant stabilization effects but also avoids the negative effect on the long-term visual appearance of paper-based relics. Even though the interaction between paper fiber and CNF film has not been analyzed, it can be assumed that hydrogen bonds constitute a significant portion of bonding forces. CNFs can fill paper gaps, increase the total number of hydrogen bonds, and reinforce paper. Moreover, Völkel et al. [42] coated the fire-damaged historical paper with a thin layer of CNFs. This method enabled the reliable preservation of damaged paper and, most importantly, retrieved the contained historical information. After treatment, CNFs form a transparent film on the surface of paper and adhere to the damaged matrix (Figure 4a). SEM images show that there is also strong adhesion to individual fibers and penetration of CNFs into larger accessible pores (Figure 4b,c). AFM analyses of the treated paper surface further confirm the homogeneous application of CNFs, which partially reduce the original substrate’s roughness (Figure 4d). Concurrently, the CNF layers reduce brittleness and re-establish a certain degree of flexibility (Figure 4e,f). Furthermore, the effectiveness of reinforcing the fragile and brittle paper by CNFs was assessed through individual bending tests. These tests revealed that CNFs enhanced the material’s resistance to bending and mechanical influences.

For centuries, iron gall ink was one of the most common used writing media. However, corrosion caused by iron gall ink has been a serious damage to the literature. The use of iron gall ink always leads to the endogenous degradation of paper through two mechanisms, namely, acidic hydrolysis of cellulose due to high acid content and oxidative degradation of cellulose associated with free iron ions [70,71]. Currently, the calcium phytate/magnesium phytate method is the most popular method to prevent corrosion of iron gall ink [72,73]. Phytate chelators are able to occupy all the coordination sites of iron and thus prevent it from participating in the Fenton reaction. Along with the antioxidant treatment of paper with phytates, the acidified paper is usually deacidified with calcium bicarbonate and calcium carbonate. This method of restoration has been widely accepted by restorers. Besides, ink corrosion is also accompanied by severe mechanical damage like cracks, fractures, and perforations, thus additional stabilization and consolidation treatments are always required. Traditional consolidation of paper relics is usually performed with thin Japanese paper (JP) and adhesives, while now CNFs have been promising alternatives. Völkel et al. [43] demonstrated the stabilizing effect of CNFs in combination with calcium phytate/Ca(HCO_3_)_2_ on damaged paper caused by iron gall ink. On the one hand, Ca(HCO_3_)_2_ was utilized for deacidification, and calcium phytate was applied to complex with iron ions. On the other hand, CNFs were used to mechanically consolidate the corroded paper. The use of CNFs does not compromise the integrity of the manuscripts and provides substantial mechanical reinforcement effects. CNFs hold considerable promise for the restoration of severely damaged manuscripts during phytate treatment. Notably, CNFs minimally affect the optical properties of the manuscripts and preserve paper haptic properties. In addition, the network of CNFs acts as a protective layer of manuscripts during accelerated aging, which provides a new idea for the treatment of paper manuscripts damaged by iron gall ink.

CNFs have been excellent reinforcing materials for historical papers. However, the direct use of CNFs in paper still has some limitations. The method of using CNF suspension spraying or impregnating paper is simple and effective, but the viscosity of CNF suspension increases rapidly with the increase in CNF concentration, and the cellulose filaments are not easy to penetrate the paper networks. To better maintain the flexibility and uniformity of thin CNF coatings, CNFs should be modified under certain conditions. Typically, CNFs are modified by physical adsorption, surface chemical modification, and polymer grafting copolymerization methods [74,75]. Recently, Li et al. [44] suggested employing reduced cellulose nanofibers (rCNFs) combined with aminopropyltriethoxysilane-modified CaCO_3_ (APTES-CaCO_3_) to maintain the integrity of aged paper. First, oxidized cellulose nanofibers (OCFs) were prepared by the TEMPO-mediated oxidation route. Since OCFs contain acidic and reductive groups, such as carboxyl groups and aldehydes, which might lead to paper acidification and even induce paper yellowing during long-term storage [76,77]. Therefore, OCFs were further reduced by sodium borohydride (NaBH_4_) to obtain the reduced oxidized cellulose fibers (rOCFs). Finally, the rOCFs suspension was sonicated to get the final rCNFs. The as-prepared rCNFs have low carboxylate content and good swell ability, resulting in superior protection ability for aged paper. To enhance the dispersion stability of CaCO_3_ in alcohol, CaCO_3_ particles were modified with APTES. The APTES-CaCO_3_ particles dispersed by ultrasound were then deposited onto the aged paper using a specialized spraying vacuum container. Subsequently, the homogenous rCNFs dispersion was also applied to the aged paper in the same manner. The treated paper, featuring both rCNFs and APTES-CaCO_3_, has an internal pH of 8.31 and an alkaline reserve of 0.8056 mol kg^−1^. Notably, its tensile strength has been enhanced by 33.6%. The artificial aging tests validate the efficacy of the rCNFs/APTES-CaCO_3_ treatment, marking it a potential candidate for long-term conservation of aged paper.

### 3.3. Application of CNCs in the Conservation of Paper-Based Cultural Relics

Compared with CNFs, CNCs are rod-shaped, transparent, and highly crystalline nanoparticles with large surface areas and abundant hydroxyl groups [78,79]. Generally, CNCs prepared by HCl hydrolysis are uncharged and electrically neutral, while CNCs prepared by H_2_SO_4_ or H_3_PO_4_ hydrolysis are negatively charged with better dispersibility. However, CNCs prepared by H_2_SO_4_ and H_3_PO_4_ hydrolysis have lower stability than CNCs prepared by HCl due to the presence of acidic groups. CNCs afford a promising reinforcement effect to paper via generating strong hydrogen bonds between CNCs and paper fibers. Considering the compatibility of CNCs with paper, CNCs have been added to paper pulp before sheet formation [80] or used as coatings for paper reinforcement [81]. Moreover, CNCs have been widely used in the conservation of paper relics.

**Figure 5:**
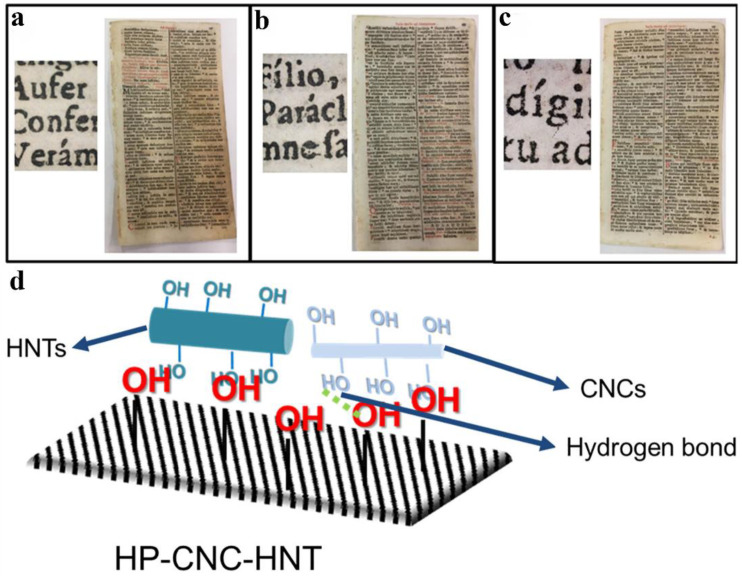
Images of pages and details from Breviarium romanum ad usum fratrum minorum treated with water (pristine) (**a**), S_CNC (**b**), and N_CNC (**c**) [45] (reproduced with permission from ACS Applied Materials & Interfaces; published by American Chemical Society, 2021). Schematic diagram representing the reinforcing process of the transparent conservative CNC-HNT layer to historic paper (**d**) [48] (reproduced with permission from Progress in Organic Coatings; published by Elsevier, 2022).

Operamolla et al. [45] proposed an innovative strategy to improve the mechanical properties of paper artwork. In their study, sulfated CNCs (named S_CNCs) prepared by sulfuric acid hydrolysis form stable colloidal suspensions in water due to surface charge repulsion. While neutral CNCs (named N_CNCs) hydrolyzed by hydrochloric acid produce uncharged nanocrystals and display higher aggregation tendency in water. S_CNCs and N_CNCs are both utilized for paper stabilization, and CNCs do not affect the visual characteristics of the aged paper (refer to Figure 5a–c). Moreover, S_CNCs can self-assemble into thin films, which uniformly cover the paper’s surface. Conversely, N_CNCs apparently bound strongly to the surface of individual paper fibers without forming self-standing films. However, the pH of paper treated with S_CNCs decreases under aging, which means the negative effect of S_CNCs on treated paper artifacts in the long term. In contrast, N_CNCs are safer for paper conservation treatment because they do not compromise the pH and mechanical properties of paper during accelerated aging. Moreover, this restoration strategy is reversible since CNCs can be removed by Gellan hydrogel with an electrochemical tool, potentially avoiding the erosion of the artifact’s integrity.

Camargos et al. [46] proposed a composite of CNCs with propylene glycol, methyl cellulose, and CaCO_3_ to fill the lacunae in paper documents and artworks. The high crystallinity index, the good stress–strain behavior, the suitable visual aspects, and the compatibility between CNCs composite material and paper guarantee the applicability of this novel method to aid the paper restoration. Later, Camargos et al. [47] combined CNFs, CNCs, and lignin nanoparticles (LNPs) in aqueous dispersions and applied the composites as protective coatings onto cellulosic substrates, that is, paper, wood, and fabric. Considering the morphological, mechanical, and structural differences between the longer, flexible CNFs and the shorter, stiffer CNCs, the CNF/CNC composite films are flexible, flatter, more uniform, and less shiny than CNC-only films. The incorporation of LNPs provides antioxidant and UV protection capacity, improving the thermal and chemical stability of the nanocomposites. These coatings effectively protect substrates against degradation under moist-heat aging and UV irradiation. The surface morphology and roughness of the substrates are well preserved after coating. Moreover, these nanocomposite layers can be easily detached from cellulosic substrates using a pHEMA/PVP chemical hydrogel, presenting an encouraging strategy for the preservation of cellulosic artifacts.

Elmetwaly et al. [48] developed a promising coating layer for the consolidation of historical paper. The coating was made by CNCs obtained from cotton fiber with a diameter of 10 nm. Then, inorganic halloysite nanotubes (HNTs) were uniformly dispersed in CNCs colloidal solution to produce a new transparent reinforcing composite for historical paper. The tensile strength of the consolidated historic paper was significantly improved. The reinforcing ability of the coating can be assigned to the strengthening of fiber bonds among historic paper and filling voids among historic paper fibers. This is attributed to the high tendency of new coating (CNC-HNT) for hydrogen bonding formation in fibers of historic paper. The rich hydroxyl groups on the surface of CNCs and HNTs and their interactions with each other lead to a high tendency for hydrogen bonding formation between them and paper fibers and then strengthen paper fiber bonds (Figure 5d). Moreover, the alignment of CNC-HNTs in different directions leads to a high degree of entanglement of the reinforcing layer with paper fibers and strengthens the fiber bonds among the paper matrix.

**Figure 6:**
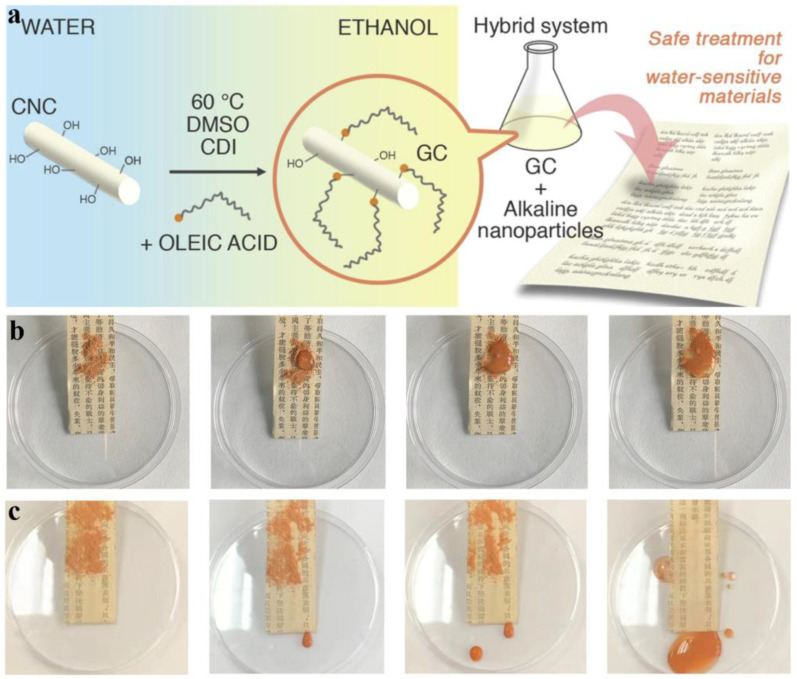
Oleic acid-grafted CNCs and alkaline nanoparticles for the strengthening and deacidification of cellulosic artworks (**a**) [49] (reproduced with permission from Journal of Colloid and Interface Science; published by Elsevier, 2020). Self-cleaning property of treated paper coated by CNC/CaCO_3_/PDMS (**b**) and original historic paper (**c**) [50] (reproduced with permission from International Journal of Biological Macromolecules; published by Elsevier, 2022).

CNCs expose large numbers of hydroxyl groups on the surface, which make them easy to be modified and combined with other substances. Xu et al. [49] grafted CNCs with oleic acid to increase their dispersibility in organic solvents and synthesized Ca(OH)_2_ or CaCO_3_ nanoparticles via a solvothermal process. The grafted CNCs and alkaline nanoparticles are used to prepare ethanol-based “hybrids” for the strengthening and deacidification of cellulosic artworks (Figure 6a). The treated paper obtains improved mechanical properties and increased pH value. The addition of alkaline nanoparticles acts synergistically to increase the interaction between the grafted CNCs and lead to the formation of CNCs clusters with increased size. The ‘‘hybrids” systems effectively facilitate the strengthening and deacidification of cellulosic materials without significant changes in the visual aspects of paper samples.

Generally, paper is vulnerable to moisture due to its hydrophilicity and porosity. In addition, paper is easily contaminated by dyes or sewage, resulting in the loss of important information on the surface. Ma et al. [50] proposed a superhydrophobic self-cleaning coating derived from CNCs coated with CaCO_3_ nanoparticles for the preservation of historic paper. First, CNC suspensions were sprayed onto historic paper samples. Subsequently, polydimethylsiloxane (PDMS)-incorporated CaCO_3_ particles were sprayed on the surface of CNCs-coated historic paper. Afterwards, historic paper was hydrophobically modified using methyltrimethoxysilane (MTMS) by chemical vapor deposition (CVD) method. The deposition of CaCO_3_ particles on the paper literature greatly improves the surface roughness of paper, and the use of MTMS with low surface energy endows historic paper with hydrophobic properties. The Si-OCH_3_ of MTMS can be hydrolyzed to Si-OH, and Si-OH condenses with hydroxyl of CaCO_3_, CNCs, and paper fibers to achieve the grafting of MTMS and the change of surface properties. The use of CNCs not only improves the mechanical strength of paper documents but also realizes the non-wetting transformation of hydrophilic hydroxyl groups, which helps to build a super hydrophobic surface on paper. As shown in Figure 6b, the soil on the original paper surface was hard to wash away, and the paper was quickly wetted and contaminated. While in Figure 6c, the dripping water washed away the soil from the surface of modified paper, thus getting the effective self-cleaning.

**Figure 7:**
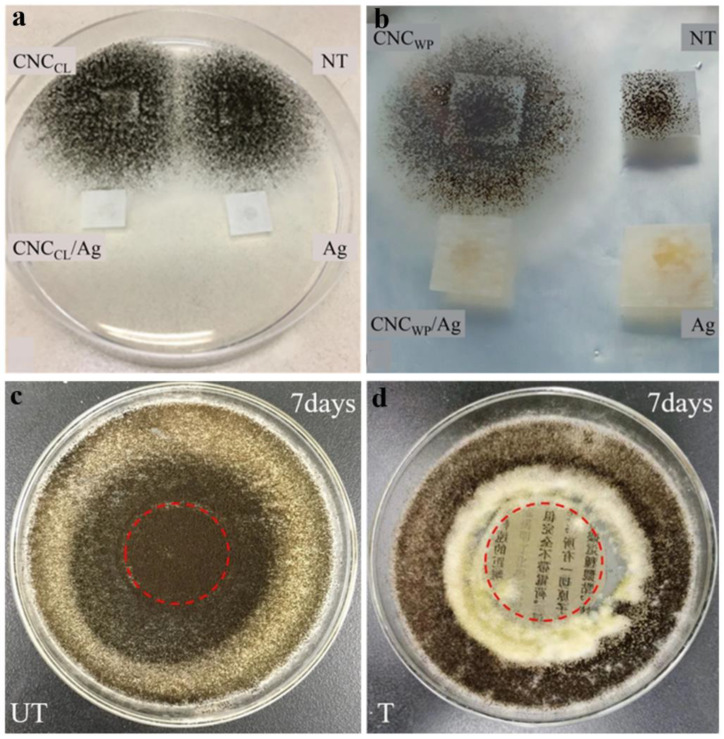
Growth inhibition of *Aspergillus niger* on Whatman paper samples, not treated (NT) and treated with CNC, CNC/Ag, and Ag nanoparticles (**a**,**b**) [51] (reproduced with permission from Carbohydrate Polymers; published by Elsevier, 2020). The 7-day anti-*Aspergillus Niger* effect of historic papers, untreated (**c**) and treated by CNC/PHMG (**d**) [53] (reproduced with permission from Cellulose; published by Springer Nature, 2021).

CNCs can also be used in combination with antimicrobial substances to provide antifungal and antibacterial activities against common microorganisms usually found in libraries, archives, or museums. For example, Bergamonti et al. [51] extracted CNCs from cotton linter and waste paper and mixed them with silver (Ag) nanoparticles for paper preservation and consolidation. Ag has a broad antimicrobial ability and strong toxicity to different microorganisms. The strengthening of paper by CNCs has been supported by mechanical tests. CNCs coating enhances the inter-fiber interactions and allows stress transfer inside the materials. The CNC-based treating method enhances the plasticity of the substrate. By adding Ag nanoparticles, it gives biocidal ability on paper against *Aspergillus niger* and would not affect the paper appearance (Figure 7a,b). Jia et al. [52] reported a simple approach to synthesize ZnO nanoparticles in CNCs by in situ solution casting. CNCs were used as effective dispersion matrices for ZnO nanoparticles. The paper coated with ZnO-CNC composites possesses antibacterial and antifungal activity against five common fungi (*Aspergillus niger*, *Aspergillus Versicolor*, *Rhizopus nigricans*, *Saccharomycetes*, and *Mucor*) observed in the archive or museum and two bacteria (*Staphylococcus aureus and Escherichia coli*) in common life. Ma et al. [53] proposed a reinforcement and mold-resistant method for ancient books based on the suspension of CNCs and polyhexamethylene guanidine (PHMG). Guanidine cationic polymers have high antifungal, antibacterial, and antiviral properties and low toxicity to humans. Moreover, the positive PHMG can form electrostatic attraction with the negative fibers, which may contribute to the formation of the hydrogen bonds between chloride ions of PHMG and hydroxyls of nanocellulose. After treated by CNCs and PHMG composites, the paper mechanical properties are significantly improved. Meanwhile, the excellent antimildew properties are observed on treated paper (Figure 7c,d). It can be a promising conservation method for ancient paper.

### 3.4. Application of BNC in the Conservation of Paper-Based Cultural Relics

Compared with CNFs and CNCs extracted from plant fibers, BNC obtained from certain bacteria species is exceptionally pure with high molecular weight, crystallinity, and hydrophilicity. BNC has a peculiar capability to self-assemble secreted fibrils into nanostructured material, and the presence of abundant hydrogen bonds within the BNC network promotes the formation of highly ordered supramolecular aggregates. BNC can be added into the pulp during the paper-making process to improve the mechanical properties of paper. For example, Xiang et al. [82] used BNC to consolidate paper made of non-woody fibers, for example, sugarcane bagasse. After mechanical disintegration, BNC with a large number of hydroxyl groups shows high bridging ability to hold paper fibers together. Skočaj et al. [83] summarized the applications of BNC in the papermaking industry, including its use (1) as a paper-reinforcing agent, (2) to improve the properties of paper made from low-grade fiber resources, (3) for application at the paper surface (as coating) in the restoration of damaged paper or to increase the barrier properties of paper, and (4) to improve fire resistance of paper. In general, the use of BNC increases the durability of the pulp when integrated into paper and improves the surface properties of paper.

For paper restoration with BNC, two methods could be used. One is the separate production of BNC layers to be subsequently applied over the document that is to be restored; the other is the direct generation of BNC on the surface of the document. In order to evaluate the possibility of BNC used for the restoration of degraded paper, Santos et al. [84] characterized the properties of BNC layers cultivated and purified by two different methods, namely, an alkaline treatment when the culture media contains ethanol (for an ex situ production of BNC layers) and a thermal treatment if the media is free from ethanol (for an intended in situ synthesis of BNC on the document surface). Results show that BNC has a high crystallinity index, low internal porosity, good mechanical properties, and high stability over time, especially when purified by the alkaline treatment. These features make BNC an adequate candidate for degraded paper reinforcement.

**Figure 8:**
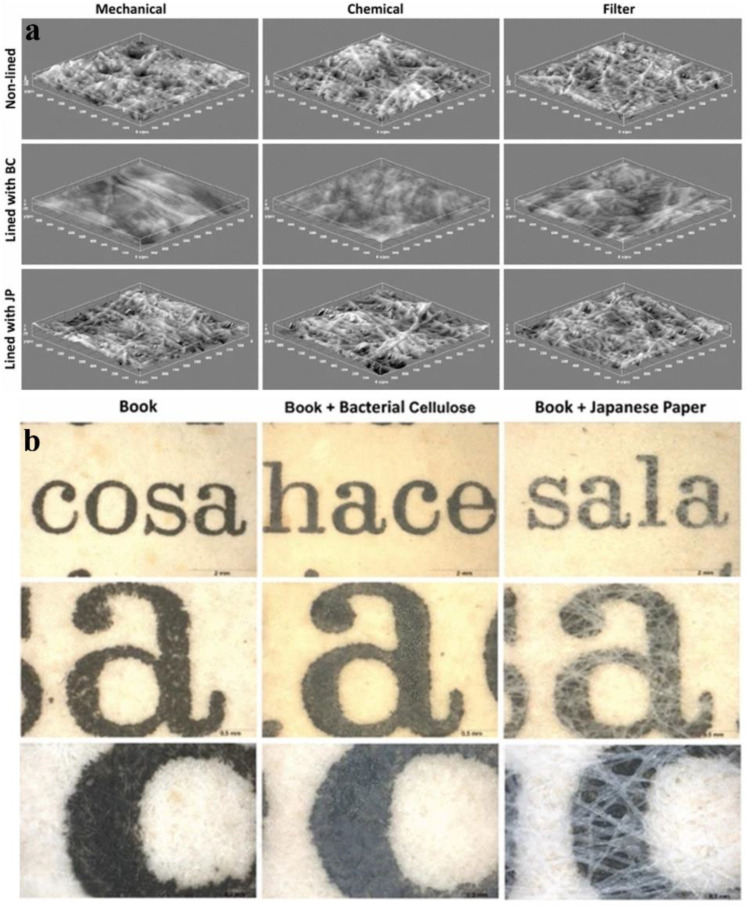
Laser profilometry (LP) images of the different non-aged samples, non-lined and lined with BNC and JP (**a**) [54] (reproduced with permission from Journal of Materials Science; published by Springer Nature, 2016). Microphotographs taken observing Book 1 with a stereoscopic microscope (first and second rows) and with an optical microscope (last row) (**b**) [55] (reproduced with permission from Journal of Materials Science; published by Springer Nature, 2016).

Later, Santos et al. [54] used BNC sheet as lining paper to restore degraded paper. Several model papers have been selected and characterized before and after lining with BNC and JP to compare the restoration capacity of both materials. The native wheat starch was used as an adhesive. The results indicate that BNC-lined papers present higher gloss values than those lined with JP since the application of BNC reduces the surface roughness of model papers significantly (Figure 8a). The wettability of BNC-lined papers decreases more markedly and independently. The closed structure of BNC leads to a reduction in air permeability, which could be beneficial to protecting papers against atmospheric pollutants. The stability over time of papers lined with BNC is proved in an accelerated aging process. The use of BNC as a reinforcing material may offer advantages for specific conservation treatments, being more suitable for certain types of paper than JP. Furthermore, BNC was used on the real samples of degraded old papers [55]. The results indicate that the mechanical properties of paper lined with BNC are as good as those obtained with JP. The modification of the optical properties is more noticeable when BNC is used as reinforced material. As shown in Figure 8b, the letters covered with BNC are totally readable, while the lining with JP fibers reduces the legibility of the texts. Moreover, the reinforcement with BNC with a closed structure results in protection of the book against atmospheric pollutants. These results suggest that BNC is a promising alternative material for the restoration of paper. They further compared the effects of BNC and JP on the visual appearance of printed paper [85]. Lining with JP notably affects the print density and chromaticity, while lining with BNC results in lower decrements in color intensity. BNC produces uniform color areas and fewer changes in color appearance. Thus, the use of BNC sheet has more advantages over JP.

Then, Santos et al. [56] deposited a thin layer of BNC (ca. 10 μm) on the paper surface by in situ cultivation for paper reinforcing. The paper modified by BNC retains its basic weight and thickness. The depositing of BNC does not enhance the paper’s mechanical strength values but keeps them after accelerated aging. The variations in color and opacity are minor after aging. The data of the static and dynamic contact angles indicate that the BNC depositing makes a more hydrophobic surface. In summary, the growth of BNC on degraded paper could be a feasible and economic option in the strengthening of paper surfaces.

In addition to the use of BNC layer as lining paper for reinforcement, BNC can be dissociated in water to get diluted suspension and directly used as a consolidant for paper repair without adding wheat starch adhesive. For example, creases and folds in Chinese paintings and calligraphy scrolls are hard to avoid during the handling in storage and exhibitions. Chen et al. [57] proposed to use a new consolidant, BNC, for the conservation of creased Chinese painting scrolls. After dissociation, the rich, free hydroxyl groups of BNC have strong bridging effects to hold paper fibers together. Xuan paper lined with BNC has a significant improvement in the folding resistances. Moreover, creased Xuan paper treated with BNC has better durability than that repaired with traditional paper strips, indicating the potential of BNC to be a promising material for the conservation of painting scrolls. Besides, Wu et al. [58] prepared stable BNC solution by dissolving BNC in NaOH/urea and proposed its application in ancient paper restoration by ultrasonic atomization. BNC is uniformly bridged on the paper fiber surface or between the paper fibers, which plays a good role in strengthening the aged paper. After treatment, the mechanical strength of the aged paper is notably improved. Even after accelerated aging for 30 days, the strength of the repaired paper was the same as or stronger than the untreated paper. This method opens a new field for the reinforcement and long-term protection of paper-based materials.

Generally, the brittle book paper is acidified and often requires deacidification while being reinforced. In order to achieve the one-step deacidification and consolidation of aged paper, Li et al. [59] prepared BNC suspensions composite with ZnO nanoparticles. BNC/ZnO nanocomposite has better thermal stability since the oxygen ions in ZnO interact with hydroxyl groups on BNC. The composite was uniformly sprayed on the paper surface. The results indicate that BNC/ZnO nanocomposites fill the gaps in paper fibers and attach to the paper surface. The mechanical strength and pH of the paper samples are significantly improved. Additionally, the antimicrobial results show that the nanocomposites have strong antifungal properties. Recently, Mou et al. [60] disintegrated and chemically modified BNC by grafting APTES to improve the interface bonding of BNC-reinforced aged paper. The stronger hydrogen bonding of APTES-BNC with the aged paper is speculated to take responsibility for the increase in paper mechanical properties. After reinforcement, the tensile index, tear index, and folding resistance of aged paper are significantly enhanced, and the pH changes from acidic to alkaline due to the amino groups of APTES-BNC. Further, they applied APTES-BNC loaded with nano magnesium oxide (MgO) to repair aged paper [61]. APTES-BNC/MgO composites can effectively remove acid from paper and enhance the strength and aging resistance of paper. Additionally, the composites are able to maintain the hydrophobicity of paper so as to prevent it from moisture. The presence of magnesium oxide nanoparticles delayed the aging process and reduced the yellowing rate of the paper.

**Figure 9:**
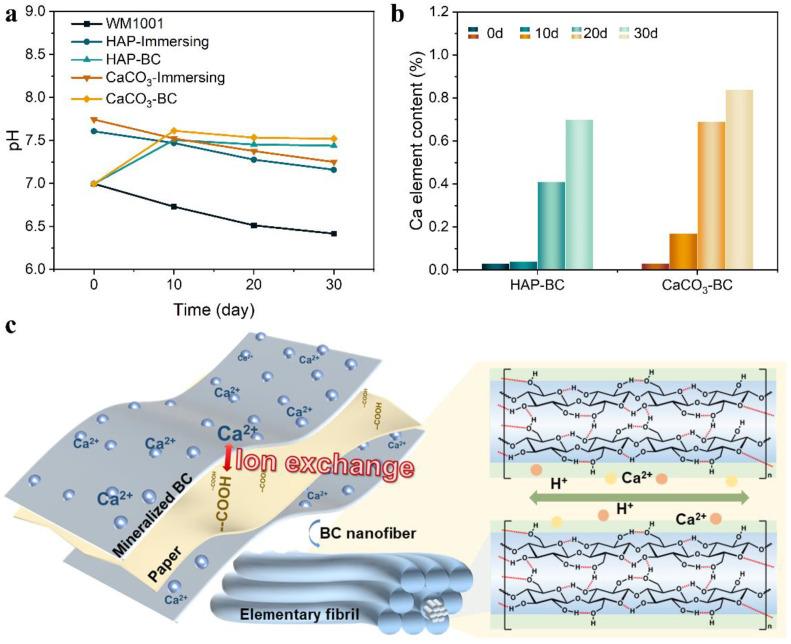
pH value (**a**), Ca element content (**b**) of paper, and the schematic of BNC deacidification mechanism (**c**) [62] (reproduced with permission from ACS Applied Materials & Interfaces; published by American Chemical Society, 2024).

Since BNC membrane has a unique porous network with abundant hydroxyl reactive sites on its nanofibers, various synthetic or chemical modifications can be easily achieved. BNC-based nanocomposites with tailored structures and designed functions can be expected to show great potential for the multifunctional protection of paper relics. Recently, Zhang et al. [62] prepared a mineralized BNC membrane for the reversible deacidification and preventive conservation of paper relics. By the enzyme-induced mineralizing, they achieved the high-loaded and homogeneous calcifications of hydroxyapatite and CaCO_3_ nanoparticles onto the BNC fibrils. The mineralized BNC membrane shows efficient and sustained deacidification performance by direct contact with paper. As shown in Figure 9a, the pH of the paper treated by mineralizing the BNC membrane clearly increased from 7.00 to about 7.50. During deacidification treatment, the Ca^2+^ ions from the BNC membrane migrate to paper and the H^+^ ions from the paper transfer to the BNC membrane by ion exchange (Figure 9b). The nanochannels of the BNC membrane offer the routes of acid–alkali neutralization and ion exchange between the BNC membrane and paper (Figure 9c), and the final paper pH values are well maintained within a certain range. Moreover, this BNC-deacidified process is reversible because BNC can be removed from paper directly. More importantly, the mineralized BNC membrane exhibits excellent thermal stability and flame-retardancy performance on paper, owing to its unique organic–inorganic composite structure. Further, they prepared BNC membrane incorporating cadmium telluride (CdTe) quantum dots (QDs) and rhodamine B (RB) fluorescent probes for the visual acidity detection in paper [86]. Based on the superior structure of BNC, this composite membrane achieves the rapid and non-destructive identification of acidification degree and acid distribution across multiple regions of acidified paper.

## 4. Conclusions and Perspectives

The excellent properties of nanocellulose afford it as an innovative consolidation material for historical paper. No need to add adhesives; CNFs and CNCs can directly stabilize and reinforce aged paper by generating strong hydrogen bond interactions between hydroxyls of nanocellulose and paper fibers. Compared with JP, BNC-lined paper presents a high gloss value and a reduction in air permeability.

The composite of nanocellulose with other functional materials greatly expands its application scope. More importantly, the rich hydroxyl groups on the large external surface of nanocellulose can be chemically modified to achieve better properties and functions. The reductive treatment of CNFs provides low carboxylate content and good swell ability. The chemical grafting of oleic acid on CNCs increases the dispersibility in organic solvents. The modification of CNCs and paper fibers by MTMS endows historic paper with hydrophobic properties and self-cleaning capability. The mineralization of BNC endows excellent deacidification and flame-retardancy functions.

Since CNFs, CNCs, and BNCs have different structural characteristics, the structure–function relationship should be emphasized during their application on paper conservation. For example, CNCs are thinner and shorter than CNFs and BNCs, which have better dispersion in solvents and can be modified and utilized more easily. Besides, the composite of nanocellulose with other materials by designed chemical modification or simply mechanical mixing should be considered. The interaction forms between nanocellulose and the incorporated materials will affect the final protection performance.

In most instances, nanocellulose in the form of suspension is sprayed or brushed onto the surface of paper for coating or voids filling, while BNC layer can be lined to paper by adhesive or produced in situ on paper or in the form of a mineralized membrane to directly contact with paper. In view of the principles of reversibility and minimal intervention in heritage conservation, some applied nanocellulose can be removed by hydrogels, while others cannot be removed from paper surfaces. More importantly, some negative effects of the applied materials should be focused on, such as the antimicrobials of Ag and ZnO. Ag is harmful to humans and the environment, and ZnO can catalyze a photochemical oxidation reaction to make paper yellow/brown discoloration. Therefore, more attention should be paid to the feasibility and safety of using nanocellulose and its composites so as to mitigate the potential risks to paper-based cultural relics.

## Figures and Tables

**Table 1:** Application of nanocellulose and its composites for the conservation of paper-based cultural relics. (Abbreviations: CNF: cellulose nanofibril; CNC: cellulose nanocrystal; BNC: bacterial nanocellulose; S_CNC: sulfated CNC; N_CNC: neutral CNC; rCNF: reduced CNF; APTES: aminopropyltriethoxysilane; PDMS: polydimethylsiloxane; MTMS: methyltrimethoxysilane; HAP: hydroxyapatite; HNT: halloysite nanotube; PHMG: polyhexamethylene guanidine; RH: relative humidity; JP: Japanese paper).

Nanocellulose and Its Composites	Forms of Nanocellulose	Types of Paper	Application Method	Purpose and Function	Current Conservation Effect	Long-Term Conservation Effect	Reference
CNF	Suspension	Rag paper; book paper without and with lignin; Whatman no. 1 paper	Applied with a brush or film applicator on a vacuum panel	Consolidation	Low optical and haptic changes; consolidation of weakened paper areas; exerting a reinforcing impact without introducing extra bonding substances such as glues	During the process of aging tests conducted in sealed vessels over periods of 6 or 20 days, the alterations observed were comparable to those in untreated samples, indicating that treated paper with CNFs do not introduce any negative effects over time.	[41]
CNF	Suspension	Fire-damaged volumes from the 18th and early 19th centuries and made of rag paper	Applied with a brush from the center of the paper, stroking outward over all damaged areas	Consolidation	Significantly minimize its fragility, enhance stability, facilitate digitization and extend handling; no negative effect on visual appearance and legibility; re-establish a proper level of flexibility, enhance resistance to bending.	In an accelerated aging test conducted at 80 °C for 38 days, all types of used CNFs effectively stabilized the charred areas of paper. After drying, no apparent differences in visual appearance are seen. The CNFs can be regarded as safe for extended use.	[42]
CNF + calcium phytate + Ca(HCO_3_)_2_	Suspension	Rag papers from a collection of handwritten sermons from the years 1839 and 1840	Applying a solution of CNFs mixed with calcium phytate or Ca(HCO_3_)_2_ to the paper surface	Deacidification, chemical stability, and consolidation	Combining deacidification, mechanical stabilization, and chemical stabilization; avoiding an additional stabilization and drying step; no optical interference and no change of the haptic properties	CNFs acting as a stabilizing network during accelerated degradation; effective at lowering the number of chain scissions after accelerated degradation	[43]
rCNF + APTES-CaCO_3_	Suspension	Naturally aged mechanical wood-pulp paper from Popular Cinema magazine published in 1981	Applied by a spraying method	Deacidification and consolidation	When the concentration of APTES-CaCO_3_ was 5 mg mL^−1^ or higher, the treated paper exhibited an internal pH of 8.31. All treated paper samples exhibited higher tensile indexes compared to untreated samples, along with a noticeable increase in brightness.	The untreated samples showed a 26.7% reduction in strength after aging at 80 °C and 65% RH for 72 h, whereas the treated samples exhibited a smaller decrease of 14.1% to 17.3%. The ΔE of treated samples remained within a proper range (<1.5) and was visually imperceptible after aging at 105 °C for 72 h.	[44]
CNC	Suspension	A written real aged paper sample from the XVIII century; Whatman filter paper	Applied by a soft brush on stripes of Whatman or aged paper samples	Consolidation	The application of S_CNCs and N_CNCs enhanced the optical and the mechanical characteristics of samples. The reversibility of this method was clearly illustrated by the simple and efficient removal of the nanocellulose using Gellan hydrogel.	Tensile strength tests showed a notable increase in maximum stress and strain values for S_CNC- and N_CNC-treated samples. Additionally, the CNC treatment maintained the optical quality of paper even after aging.	[45]
CNC + propylene + glycol + methylcellulose + CaCO_3_	CNC-based paper pulp	Two wood cellulose paper sheets from a book printed in the 20th century	Combined compounds in aqueous medium	Consolidation	The crystallinity of CNC-based sample was triple higher than that of samples made from wood cellulose and reprocessed cellulose fibers.		[46]
CNC + CNF + lignin	Suspension	White cotton paper	Applied by brushing to form coating films	Antioxidation and UV resistance, barrier properties	Surface morphology, roughness, and substrate vapor permeability were mainly preserved after the coating application. The wettability of nanocomposite-coated substrates could be tailored and reduced to produce hydrophobic surfaces. Nanocomposite coatings can be easily removed by using waterborne hydrogels.	Under moist-heat aging and UV radiation, the nanocomposite coatings minimized the color changes in paper. Self-standing nanocomposite films with analogous formulations exhibited excellent colorimetric stability when aged under harsh conditions.	[47]
CNC + HNTs	Suspension	Real written historical paper produced in 1943 obtained from private collection	Applied by spraying the colloidal conservation solutions to paper	Consolidation and UV resistance	The tensile strength and UV protection ability of the consolidated historic paper are significantly improved. The coating layer affords outstanding transparency with good optical properties of coated paper.	Compared to the untreated sample, the coating got the consolidation of paper by twice in tensile strength after aging. The transparent coating maintained its optical integrity without any significant alterations after optical aging.	[48]
Oleic acid-grafted CNC + Ca(OH)_2_/CaCO_3_	Suspension	Filter paper (qualitative grade from cotton linters, Munktell)	Applied by brushing on both sides of paper samples	Deacidification and consolidation	The hybrid systems efficiently enhance paper strength and neutralize acidity in damaged paper, preserving the samples’ original visual qualities.	The yellowness index rose from 4 to 22 after aging, with only a slight reduction in approximately 2–3 units observed from the use of grafted CNC.	[49]
CNC + CaCO_3_ + PDMS + MTMS	Suspension	Historic paper samples taken from an old wheat straw pulp book that was published in 1954	Spraying the coatings by an AnestIwata W-71 spray gun	Deacidification, consolidation, and hydrophobization	The paper’s pH rose to a range of 7.50 to 7.7, and the alkaline reserve reached 0.677 to 1.235 mol kg^−1^. The tear strength of paper increased by 26.51%. The surface information of the paper was still clearly visible. The hydrophilic hydroxyl groups of paper were converted to hydrophobicity.	After being aged at 80 °C and 65% RH for 72 h, the reduction in tensile and tear strength of the aged paper after treatment was less than that of untreated paper. The whiteness and ΔE of treated paper were not different from that of the untreated paper.	[50]
CNC + Ag	Suspension	Whatman paper	Applied by brushing on Whatman paper in 3 passes with an interval of 20 min from each other	Antimicrobial and consolidation	Preventing the growth of *Aspergillus niger* fungus; enhancing the flexibility and durability of the paper substrate; no impact on the visual appeal of the original paper		[51]
CNC + ZnO	Suspension	The school newspapers of Renmin University of China (1960)	Sprayed twice and dried at room temperature	Antimicrobial, UV resistance, and consolidation	Enhanced tensile and folding properties of ZnO/CNC-coated paper versus uncoated paper, showing the best activity against five common fungi and two bacteria and assessing the favorable color retention in paper treated with ZnO/CNC composite	The treated samples demonstrate enhanced thermal and UV resistance, along with a reduced decline in paper durability.	[52]
CNC + PHMG	Suspension	Whatman paper; an old book made of wheat straw pulp and published in 1954	Applied by spraying with the spray gun	Antimicrobial and consolidation	An increase in folding strength (1.78 times), tearing strength (29.93%), and tensile strain (46.62%); the growth trace of mold is less than 10% in the treated historic paper	The strength of the treated sample was higher than that of the untreated sample after aging. The treatment barely altered the appearance of the historical paper, with only a faint change in brightness after being aged for nine days.	[53]
BNC	Layer	Filter paper Whatman no. 42; handsheets from mechanical and chemical pulps	As lining paper in place of JP	Consolidation, reducing air permeability	BNC-lined papers present improved mechanical properties, higher gloss values, and a more marked decrease in wettability related to the reduction in air permeance.	After aging, no significant changes were observed in any properties except for the b* and L* color parameters. The wettability rate decreased in all cases, indicating that papers lined with BNC remained stable over time.	[54]
BNC	Layer	Three books from 1940 to 1960	As lining paper in place of JP	Consolidation and reducing air permeability	BNC-lined papers had improved mechanical properties, more marked modifications on optical properties, better legibility of letters, and higher changes in porosity to protect documents from humidity and atmospheric pollutants.	The aging caused a minor decrease in burst index. Variations in tear index and optical characteristics during aging varied depending on the paper’s restoration needs.	[55]
BNC	Layer	Filter paper Whatman no. 42; handsheets from mechanical and chemical pulps	In situ growth on paper surface	Consolidation and reducing air permeability	The modified papers maintain their thickness and their basic weight, make minor changes in optical properties, inhibit the airflow through the papers, and make the surface more hydrophobic.	After aging, the mechanical characteristics of the papers were preserved. The changes in opacity and colorimetric coordinates of the reinforced papers were minor even after further aging.	[56]
BNC	Suspension	Creased Chinese Xuan paper	Applied as lining paper	Consolidation	Improving tensile index, folding resistance, and tear resistance; remaining pH value and the crystallinity unchanged; making the creased paper smoother	After exposure at 80 °C and 65% RH, the tensile index, folding resistance, and pH value of BNC-treated paper increased.	[57]
NaOH/urea BNC	Solution	An old book in 1972	Applied by ultrasonic atomization	Deacidification and consolidation	The tear index enhanced by 49.46%, while the folding endurance increased by 115%. The pH level rose to 8.12, and the alkaline reserve was 0.4 mol kg^−1^.	After aging at 80 °C and 65% RH for 30 days, the material maintained approximately 50% of its original strength.	[58]
BNC + ZnO	Suspension	The Journal of Xinhua Daily published in 1980	By spraying BNC/ZnO dispersion on both sides with an air compressor	Antimicrobial and consolidation	Improvement in tensile strength, folding endurance, the pH value, and whiteness of paper; preventing mold growth		[59]
APTES-BNC	Suspension	An old book made from bleached hardwood pulp in the 1970s	Applied by impregnating in BNC dispersion	Deacidification and consolidation	Increased tear index (87.89%), tensile index (92.96%), folding endurance (304%), and pH value (from 6.47 to 8.03); a slight change in whiteness; maintained paper hydrophobicity to prevent it from moisture		[60]
APTES-BNC + MgO	Suspension	An old book made of bleached hardwood pulp and published in 1972	Applied by impregnating in BNC/MgO dispersion	Deacidification and consolidation	An increased zero-span tensile strength (13.91%) and tensile stress (88.60%); the significantly increased pH value and alkaline reserves surpassing 0.30 mol kg^−1^	After aging, the treated paper contained a mild alkali (pH 7.56 with an alkali reserve of 0.25 mol kg^−1^) and exhibited a 32.18% increase in the tear index compared to the untreated paper.	[61]
HAP/ CaCO_3_-BNC	Membrane	Whatman paper	By direct contacting with paper	Deacidification and flame retardancy	Efficient deacidification performance; excellent flame-retardancy performance; reversible deacidification to preserve the original structure and fiber morphology of paper	The treated paper exhibits a smaller reduction in DP and a lesser increase in carbonyl and carboxyl groups during aging. The final pH of paper is stabilized in a range of 7.3 to 8.1, indicating a durable stability of paper pH.	[62]

## Data Availability

Not applicable.
